# *MysteryMaster*: scraping the bottom of the barrel of barcoded Oxford nanopore reads

**DOI:** 10.1186/s12859-025-06266-2

**Published:** 2025-10-01

**Authors:** Abdolrahman Khezri, Sverre Branders, Anurag Basavaraj Bellankimath, Jawad Ali, Crystal Chapagain, Fatemeh Asadi, Manfred G. Grabherr, Rafi Ahmad

**Affiliations:** 1 Department of Biotechnology, University of Inland Norway, Holsetgata 22, 2317 Hamar, Norway; 2 Institute of Clinical Medicine, Faculty of Health Sciences, UiT - The Arctic University of Norway, Hansine Hansens veg 18, 9019 Tromsø, Norway

**Keywords:** Oxford nanopore sequencing, Unclassified reads, Barcoding, Demultiplexing, Dorado, Guppy, Mysterymaster

## Abstract

**Background:**

The high error rate associated with Oxford Nanopore sequencing technology adversely affects demultiplexing. To improve demultiplexing and reduce unclassified reads from nanopore sequencing data, we developed *MysteryMaster*, a demultiplexer that utilizes the optimal sequence aligner, Cola.

**Results:**

When compared to Oxford Nanopore´s Dorado and Guppy demultiplexing tools across three datasets of 37 diverse samples with established ground truth, we found that *MysteryMaster* accurately identifies a similar or greater percentage of reads among the different basecalling models: Fast, HAC, and SUP. *MysteryMaster* performs slightly better than the other tools on data that was basecalled using the Fast basecalled model, while its performance in HAC and SUP data is similar to Dorado’s. *MysteryMaster* has a false positive rate of just 0.41% with default settings.

**Conclusions:**

While *MysteryMaster* can function as a standalone demultiplexer tool, the sequential application of Dorado and *MysteryMaster* produced the best overall performance.

**Supplementary Information:**

The online version contains supplementary material available at 10.1186/s12859-025-06266-2.

## Introduction

Oxford Nanopore Technology (ONT) is a popular sequencing technology recognized for its cost-effectiveness, ability to generate long reads, and the integration of real-time sequencing with data analysis capabilities. Following recent developments, ONT now allows sequencing of up to 96 samples in a single run, which is highly valuable for single-cell sequencing or experiments under different conditions. To sequence multiple samples, each must be tagged with a short, unique barcode. These barcodes are also sequenced and play a crucial role in accurately assigning reads to the correct samples. ONT is also known for its high error rate, although this was significantly reduced after the launch of the R10.4.1 flowcell compared to the R9.4.1 technology [1, 2]. Sequencing errors and the library preparation process can prevent some reads from being correctly assigned to their respective barcodes. This leads to a large portion of unclassified reads, which can reach up to 20% [3, 4]. This causes the loss of sequencing data, impacting downstream results, increasing costs, and requiring additional effort for sample preparation.

To get the most out of the multiplexing capability of ONT, an efficient barcoding solution is necessary to demultiplex this data. The solution was initially provided within the Guppy pipeline and is now integrated with the new Dorado software by ONT. Likewise, adapter trimming tools like Porechop [5] and HycDemux [6] also allow for demultiplexing ONT reads, but they were developed for the outdated and discontinued ONT 9.4 chemistry and Rapid barcoding kits. Recently developed tools such as Flexiplex [7], BLAZE [8], and scTagger [9] performed well for demultiplexing using both real and simulated datasets of different sizes. However, to troubleshoot the demultiplexing problem in ONT data effectively, an ideal tool should allow users to visually verify whether barcodes are present or absent in the reads, aiding in debugging.

Here, we introduce *MysteryMaster*, a software specifically designed to detect short sequences and barcodes despite the presence of insertion and deletion errors. *MysteryMaster* integrates the local sequence aligner Cola [10], which uses a non-linear scoring scheme to find alignments. This is particularly helpful for aligning brief sequences with gaps. For example, suppose two consecutive matches are on either side of a gap, and their scores exceed four nucleotides with two mismatches in between. In that case, the alignment can still be accurately determined. This approach addresses the common error patterns observed in ONT reads, which usually involve insertions and deletions.

## Software and implementation

### Workflow

*MysteryMaster* can take either a fastq file or a directory of files with the Fastq or fq.gz extension as input. It also needs a FASTA file with barcodes, which is freely configurable and can contain any set of custom barcodes, yet two default options (24 and 96 barcodes) are available in the git repository. The tool processes each read in either single- or multi-threaded mode, producing two FASTQ files for every barcode: one with adapter-trimmed reads and another that includes trimmed sequences along with the barcodes, as well as the trimmed-off adapter sequences for additional quality control (QC) assessments. To ensure proper trimming and the usability of the remaining sequence for analysis, *MysteryMaster* allows discarding reads shorter than a threshold (defaulting at 550 nucleotides), which are saved to a separate file. Moreover, it outputs all unclassified sequence reads and generates a summary table with statistics for each barcode and the unclassified sequence reads.

### Algorithm

*MysteryMaster* is built around the Cola aligner [10], which is highly configurable and can find short alignments in the presence of insertions, deletions, and mismatches. Initially, each barcode is aligned with the first 110 nucleotides of every read. Subsequently, the alignments are ranked according to the Smith-Waterman (SW) score, necessitating that the highest-scoring alignments exceed the second-best-scoring alignment by a margin of 2 or more to resolve potential ties. The alignment with the highest score is then subjected to a positional filter and may be disregarded based on a combination of the SW score and alignment position to minimize false positives (refer to the following section). We also note that reads should not be trimmed before processing.

### Specificity and positional filtering

According to the ONT barcode kit specification, the barcode is located before the adapter and the genomic sequence, starting around position 47. We thus define valid windows for the alignments depending on the SW score of the alignment: for a SW score of 13, which is the default lower cutoff, alignments are by default limited to starting between positions 36 and 51; for a SW score of 14, this window is between 30 and 75 nucleotides, whereas higher scoring alignments may be located between positions 5 and 85. We determined these parameters by processing a negative control using a bacterial mock community (01_Mock_100000-bacteria-l1000-q10.fastq) compiled by Marić et al. [11] And downloaded from https://zenodo.org/records/7213115. This data set consists of 100,000 nanopore reads exceeding 1,000 nucleotides in length. As this is a composite data set with some barcoded reads that were not trimmed, we eliminated the first 150 nucleotides from each read. Out of the 100,000 reads, *MysteryMaster* accurately recognized 99,590 as unclassifiable, while 410 were incorrectly assigned barcodes based on the previously mentioned parameters. Using these parameters as defaults resulted in a total false positive rate of 0.41%, or 0.017%, for each of the 24 tested barcodes. The same analyses yielded false positive rates of 0.014% and 0.001% for Dorado and Guppy, respectively.

### Graphical user interface

*MysteryMaster* is available in two formats: (a) as a standalone executable designed for integration into pipelines, and (b) through a graphical user interface (GUI). The standalone option is more efficient for batch processing. At the same time, the graphical interface is better suited for parameter experimentation and troubleshooting, allowing for quick (re-)analysis of smaller data sets or subsets.

**Fig. 1:**
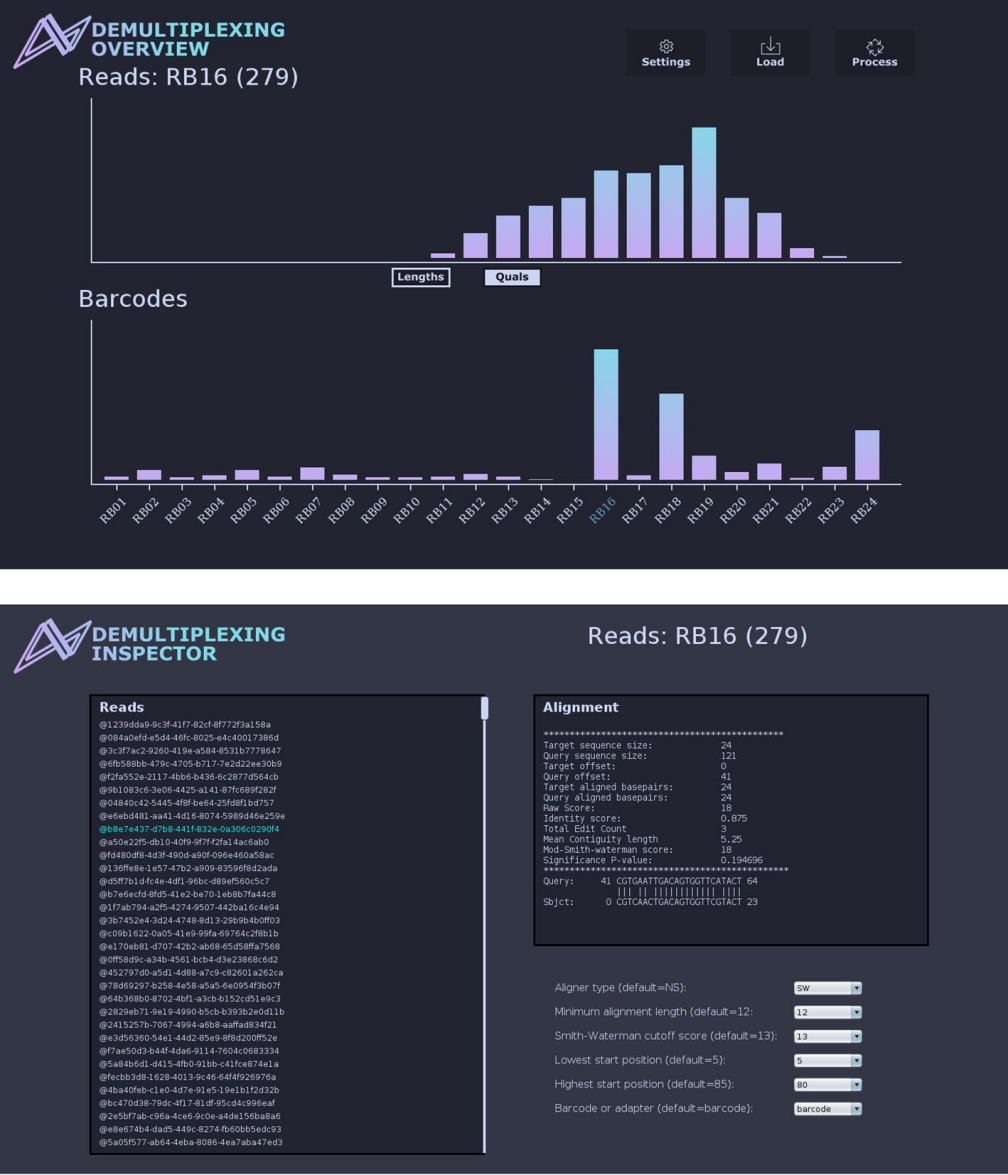
Screenshots of *MysterMaster’s* user interface showcase its features. The top panel provides an overview, allowing users to view counts for all barcodes, read lengths, and quality score distributions. Moving the mouse over the barcodes displays statistics for the unclassified reads. Below, the Demultiplexing Inspector panel illustrates barcode alignments along with their respective statistics. As alignments are computed in real time, modifying parameters in the settings panel allows for optimizing values for upcoming runs

The GUI has two views, the *Demultiplexing Overview* and the *Demultiplexing Inspector* (Fig. 1). In the Overview, statistics about barcode distributions are displayed, while the Inspector view allows changing parameters, which take effect immediately when clicking on individual reads.

## Results

To evaluate *MysteryMaster*’s performance against other demultiplexing tools, notably Guppy and Dorado, we generated data from 37 samples across three different sequencing runs using the SQK-RBK114.24 sequencing kit on FLO-MIN114 flow cells. This data contains 14 different known pure bacterial isolates, 11 urine samples spiked with uropathogenic bacteria, six blood samples spiked with sepsis-relevant bacteria, four milk samples from cows diagnosed with mastitis, and two environmental wastewater samples (Supplementary material 1). All sequencing runs and downstream bioinformatic analyses were conducted on a Dell Precision 5820 Tower workstation equipped with an Intel^®^ Xeon^®^ W-2265 CPU (3.50 GHz, 12 cores, 24 threads), 128 GB of RAM, and an NVIDIA RTX A4000 GPU with 16 GB of dedicated memory.

For all runs, all raw pod5 files generated by MinION were collected and re-basecalled using the Dorado v0.9.0 basecaller with the --no-trim option enabled in three sensitivity modes: Fast, High Accuracy (HAC), and Super Accuracy (SUP). In all conditions, the latest basecalling model (version 5.0.0) was utilized. The basecalled data (passed reads) were filtered by the nanoq tool [12], and reads shorter than 550 bp were discarded before barcoding.

In the first run (average-sized dataset), Fast, HAC, and SUP modes resulted in 661,302, 676,368, and 694,978 passed reads, respectively. The second run (small size dataset) produced 84,761, 91,313, and 97,368 passed reads for Fast, HAC, and SUP modes, respectively. The third run was the most extensive dataset tested in this study, consisting of 1,436,593, 1,456,125, and 1,478,276 passed reads for Fast, HAC, and SUP modes, respectively (see Supplementary Material 2 for further read statistics).

**Fig. 2:**
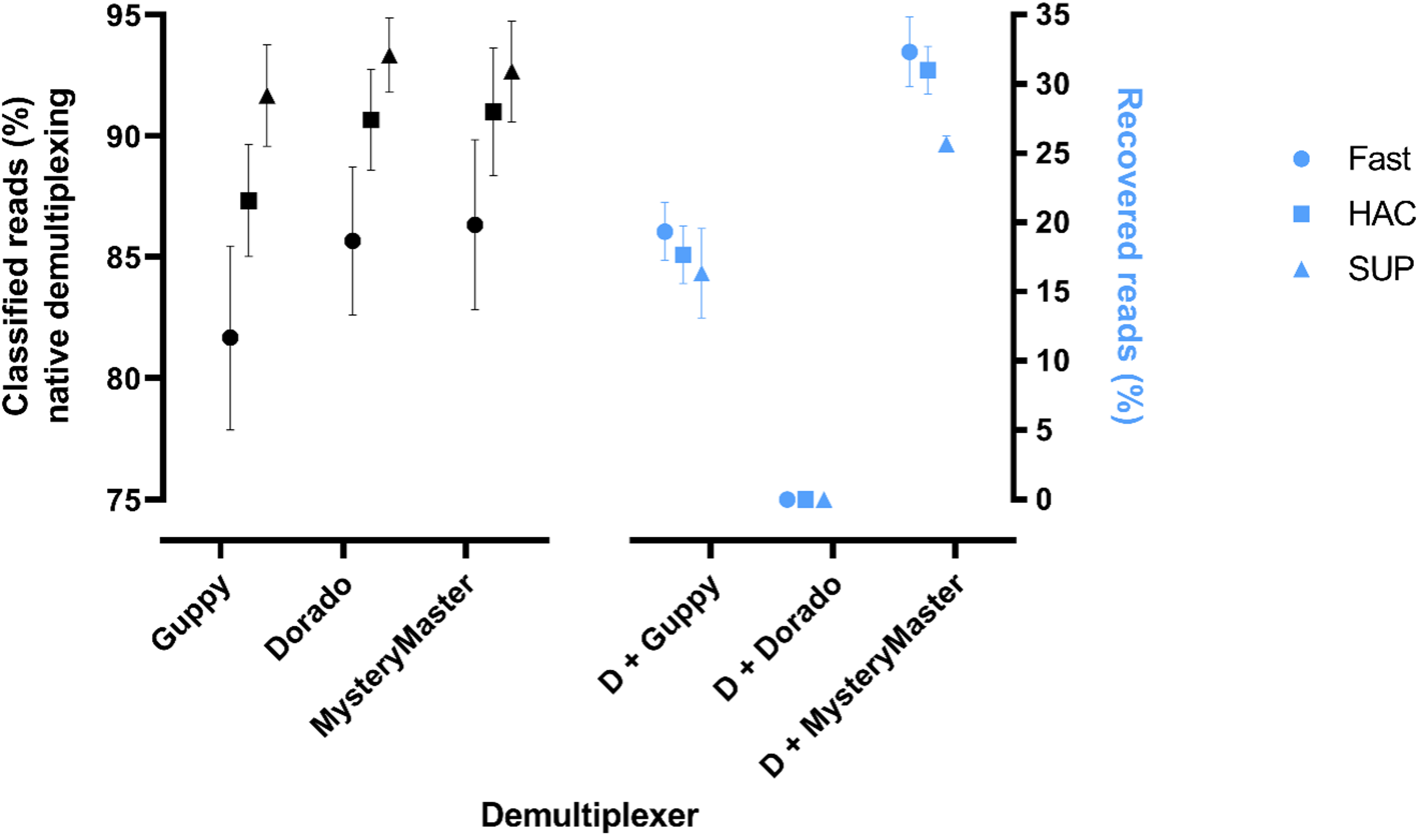
Overview of the performance of *MysteryMaster*, Dorado, and Guppy demultiplexers. Left panel: The performance of the tools to demultiplex the reads, which were base-called using three sensitivity modes (native demultiplexing): FAST, High Accuracy (HAC), and Super Accuracy (SUP). Right panel: The performance of different demultiplexers in recovering originally reported unclassified reads by Dorado in different basecalling modes, “e.g. D + Guppy indicated that Guppy was run on unclassified reads which Dorado reported”. Data is presented as mean ± SD (standard deviation)

In our initial benchmarking run (native demultiplexing), we assessed the performance of different barcoders by demultiplexing filtered reads using Guppy v6.5.7, Dorado v0.9.0, and *MysteryMaster*, all with their default settings (except for *MysteryMaster*, which was set to 20 cores). During the second benchmarking run, we focused solely on re-barcoding the unclassified reads reported by Dorado to determine if we could recover those reads. For native demultiplexing, *MysteryMaster* demonstrated a slight edge over the other barcoders when processing Fast base-called reads, with classification rates of 87%, 86%, and 82% for *MysteryMaster*, Dorado, and Guppy, respectively. Meanwhile, *MysteryMaster* showed slightly improved performance over Dorado on HAC data, but slightly lower performance on SUP data (see Fig. 2, left panel). By sequentially running Dorado and then *MysteryMaster* (in the second benchmarking run), we observed an additional reduction in unclassified reads (Fig. 2, right panel) by as much as 33%.

We also verified the Dorado barcoded reads (only base-called reads in SUP mode) for pure isolates from the first and second runs, which were demultiplexed using MysteryMaster. The results indicated that Dorado and MysteryMaster agree on read classification by an average of 98.19% and 97.09% in runs 1 and 2, respectively. Reads that were conflicted—meaning classified by Dorado but not by MysteryMaster for the same barcode—were reported as unclassified reads using MysteryMaster (see Supplementary Material 2).

Our computational benchmarking of native demultiplexing indicated that both Dorado and Guppy completed demultiplexing in approximately the same wall-clock time, with a maximum of 2 min. In contrast, MysteryMaster required more time, with a maximum of roughly 19 min observed for Run 3 (SUP basecalled data). In terms of memory usage, Guppy was the most memory-intensive demultiplexer, followed by MysteryMaster and then Dorado (Supplementary material 2).

## Discussion

Multiplexing genomic data by identifying samples via barcodes facilitates the simultaneous sequencing of multiple samples within a single operational run. Prior research has predominantly concentrated on the performance evaluation and benchmarking of base-calling algorithms [13–15]. However, it is essential that the demultiplexing step accurately assigns the sample ID by identifying the correct barcode for as many reads as possible. While mapping the unclassified reads to the anticipated genome(s) is recommended to retrieve these reads [3]. This approach can be labor-intensive, demands bioinformatics proficiency, and is inapplicable to metagenomic sequencing, where both the genome and organism remain unidentified.

The benchmarking and improvement of the current demultiplexer for ONT long reads have not been adequately addressed. Our research reveals that *MysteryMaster*, which employs the Cola algorithm to rank consecutive alignment matches non-linearly, performs comparably or even better than Dorado in its native barcode calling. Utilizing both Dorado and *MysteryMaster* yields the best overall results, as each method reveals barcodes that the other may overlook. We intend to investigate these differences more thoroughly in future studies.

Notably, *MysteryMaster* is highly configurable and features a user-friendly GUI for quickly testing various parameter settings and examining the quality of the barcode calls and reads. Specifically, the Demultiplexing Inspector panel facilitates the analysis of individual reads, allows for dynamic parameter changes, recomputes individual calls in real-time, and enables batch calling of the entire dataset from within the GUI. In our test using simulated data from a known set of barcodes (results not shown), over 99% of reads were flagged as unclassified by all three tools. However, because MysteryMaster allows users to fine-tune settings based on visual inspection of the reads to identify the barcodes, a higher percentage of simulated reads (0.72% compared to 0.37% and 0.38% for Guppy and Dorado, respectively) were later assigned to the expected barcodes. The results of this study not only demonstrated the effectiveness of *MysteryMaster* but also highlighted its ability to assist users in troubleshooting issues with unclassified reads.

In addition to comparing demultiplexers, our results indicated that the number of unclassified reads decreased as the sensitivity of base calling increased from Fast to SUP. Although the reduction in unclassified reads was slight from HAC to SUP, the results were somewhat anticipated, making this study the first to report this finding.

While the recent ONT error rate using R 10.4.1 technology is below 2% [16], a further reduction in error rates, which may mitigate the challenges associated with classifying reads, is anticipated. At the same time, we acknowledge that error rates constitute only one of the obstacles to achieving accurate classification. To facilitate ONT users in thoroughly “debugging” their data sets, *MysteryMaster* prioritizes user-friendliness and interactivity, ensuring accessibility for individuals lacking expertise in bioinformatics. Consequently, we assert that by recovering data from unclassified reads, *MysteryMaster* will help reduce costs per run, facilitate data analysis, and elucidate the complexities surrounding the unclassified reads.

## Supplementary Information

Below is the link to the electronic supplementary material.

Supplementary Material 1

Supplementary Material 2

## Data Availability

Unclassified reads used for benchmarking in this study have been deposited in the NCBI Sequence Read Archive (SRA) under BioProject accession number PRJNA1268756. Project name: MysteryMaster; Project home page: https://bitbucket.org/NPC239/mysterymaster/src/main/ and https://anaconda.org/bioconda/mysterymaster/files; Operating system(s): Linux; Programming language: C++ and Java (GUI); Other requirements: Java 11 or higher; License: GNU GPL 3; Any restriction to use by non-academics: none other than specified by the GPL 3 license.
