# Supplementary material for: MysteryMaster: scraping the bottom of the barrel of barcoded Oxford nanopore reads
Source: BMC Bioinformatics. 2025 Oct 1;26:235. doi: 10.1186/s12859-025-06266-2 (PMC12487470; doi:10.1186/s12859-025-06266-2)
Supplement: Supplementary file 2 — Supplementary Material 2 [file 12859_2025_6266_MOESM2_ESM.docx]

***MysteryMaster*: Scraping the bottom of the barrel of barcoded Oxford Nanopore reads**

Abdolrahman Khezri^1^, Sverre Branders^1^, Anurag Basavaraj Bellankimath^1^, Jawad Ali^1^, Crystal Chapagain^1^, Fatemeh Asadi^1^, Manfred G. Grabherr^1^, and Rafi Ahmad^1,2*^

1. Department of Biotechnology, University of Inland Norway, Holsetgata 22, 2317, Hamar, Norway
2. Institute of Clinical Medicine, Faculty of Health Sciences, UiT - The Arctic University of Norway, Hansine Hansens veg 18, 9019, Tromsø, Norway

* Corresponding author

**Supplementary Note**

# **1. Sample collection, DNA extraction and sequencing**

This study used pure bacterial isolates^1^, bacterial spiked blood & urine samples, clinical bovine mastitis milk samples, and water samples from a local wastewater treatment plant (WWTP) (Supplementary Table 1). Blood and urine samples were spiked with *E. faecalis,* as described previously^2,3^. The Norwegian dairy company TINE provided culture-positive bovine mastitis samples. The wastewater samples were collected from the inlet of the HIAS WWTP in Ottestad, Norway.

Supplementary Table 1 also gives an overview of the kits and methods used for DNA isolation. The isolated DNA was quantified using Qubit fluorometer 4.0 (ThermoFisher Scientific, USA), and quality was assessed using Nanodrop.

Before sequencing, DNA was purified using AMPure XP beads (Beckman Coulter, USA). The Rapid Barcoding Kit 24 V14 (SQK-RBK114.24) was used to prepare the library. All samples were sequenced using MinION flow cells 10.4.1 (FLO-MIN114) for 72 hours.

**Supplementary Table 1:** An overview of sequencing runs, samples, bacterial load, and methods for DNA extraction.

| **Sequencing** | **Sample** | **Target microorganism** | **CFU/mL** | **DNA extraction kit** |
| --- | --- | --- | --- | --- |
| Run 1 and 2 | Spiked urine  (n = 3) | *E. faecalis* | 10^5^ | Blood & Tissue kit  (Qiagen, Germany)  NAxtra^TM^ Blood total nucleic acid extraction kit  (Lybe Scientific, Norway) |
|  | Mastitis milk  (n = 4) | *S. aureus* | 10^8^ | MolYsis™ Complete5  (Molzym GmbH & Co. Germany)  Blood & Tissue kit  (Qiagen, Germany) |
|  | Pure isolate  (n = 11) | *B. cereus, M. luteus, M. catarrhalis, P. aeruginosa, S. marcescens, S. saprophyticus, S. pneumoniae, S. agalactiae, S. epidermidis, S. pyogenes, V. natriegens* | - | MolYsis Plus kit  (Molzym GmbH & Co. Germany)  Blood & Tissue kit  (Qiagen, Germany) |
|  | Inlet wastewater  (n = 2) | - | 10^5^ | FastDNA™ SPIN Kit for Soil  (MP Biomedicals, USA) |
| Run 3 | Spiked urine  (n = 4) | *E. faecalis* | 10^5^ | Blood & Tissue kit  (Qiagen, Germany)  NAxtra^TM^ Blood total nucleic acid extraction kit  (Lybe Scientific, Norway) |
|  | Spiked blood  (n = 6) | *E. faecalis* | 10^5^ - 10^7^ | QIAamp BiOstic Bacteremia Kit (Qiagen, Germany)  MolYsis™ Complete5  (Molzym GmbH & Co. Germany) |
|  | Pure isolate  (n = 3) | *B. subtilis, P. aeruginosa, S. aureus* | - | MolYsis Plus kit  (Molzym GmbH & Co. Germany)  Blood & Tissue kit  (Qiagen, Germany) |

# **2. References**

1 Ali, J. *et al.* Multi-excitation Raman Spectroscopy Complements Whole Genome Sequencing for Rapid Identification of WHO Priority Pathogenic Bacteria. *Manuscript under preperation* (2025).

2 Bellankimath, A. B. *et al.* Culture and amplification-free nanopore sequencing for rapid detection of pathogens and antimicrobial resistance genes from urine. *European Journal of Clinical Microbiology & Infectious Diseases* (2024). <https://doi.org/10.1007/s10096-024-04929-1>

3 Ali, J., Johansen, W. & Ahmad, R. Short turnaround time of seven to nine hours from sample collection until informed decision for sepsis treatment using nanopore sequencing. *Sci Rep* **14**, 6534 (2024). <https://doi.org/10.1038/s41598-024-55635-z>
